# Use of Complementary and Alternative Medicine (CAM) as Part of the Oncological Treatment: Survey about Patients’ Attitude towards CAM in a University-Based Oncology Center in Germany

**DOI:** 10.1371/journal.pone.0165801

**Published:** 2016-11-03

**Authors:** Kerstin A. Kessel, Sabrina Lettner, Carmen Kessel, Henning Bier, Tilo Biedermann, Helmut Friess, Peter Herrschbach, Jürgen E. Gschwend, Bernhard Meyer, Christian Peschel, Roland Schmid, Markus Schwaiger, Klaus-Dietrich Wolff, Stephanie E. Combs

**Affiliations:** 1 Department of Radiation Oncology, Technical University of Munich (TUM), Ismaninger Straße 22, Munich, Germany; 2 Institute for Innovative Radiotherapy (*i*RT), Helmholtz Zentrum München, Ingolstädter Landstraße 1, Neuherberg, Germany; 3 Onkologisches Zentrum im RHCCC am Klinikum rechts der Isar, Technical University of Munich (TUM), Ismaninger Straße 22, Munich, Germany; 4 Department of Otorhinolaryngology, Technical University of Munich (TUM), Ismaninger Straße 22, Munich, Germany; 5 Department of Dermatology and Allergy Biederstein, Technical University of Munich (TUM), Biedersteiner Straße 29, Munich, Germany; 6 Department of Surgery, Technical University of Munich (TUM), Ismaninger Straße 22, Munich, Germany; 7 Roman-Herzog-Krebszentrum Comprehensive Cancer Center (RHCCC), Technical University of Munich (TUM), Trogerstraße 26, Munich, Germany; 8 Department of Urology, Technical University of Munich (TUM), Ismaninger Straße 22, Munich, Germany; 9 Department of Neurosurgery, Technical University of Munich (TUM), Ismaninger Straße 22, Munich, Germany; 10 3rd Department of Internal Medicine, Technical University of Munich (TUM), Ismaninger Straße 22, Munich, Germany; 11 2nd Department of Internal Medicine, Technical University of Munich (TUM), Ismaninger Straße 22, Munich, Germany; 12 Department of Nuclear Medicine, Technical University of Munich (TUM), Ismaninger Straße 22, Munich, Germany; 13 Department of Oral and Maxillofacial Surgery, Technical University of Munich (TUM), Ismaninger Straße 22, Munich, Germany; University of Colorado Denver, UNITED STATES

## Abstract

**Introduction:**

To understand if and which patients would be open-minded to Complementary and Alternative Medicine (CAM) use parallel to their oncological treatment. Moreover, we sought to determine which methods are most accepted and which are the primary motivators to use CAM.

**Methods:**

We developed and anonymously conducted a questionnaire for patients in the oncology center (TU Munich). Questions focus on different CAM methods, previous experiences, and willingness to apply or use CAM when offered in a university-based setting.

**Results:**

A total of 171 of 376 patients (37.4% women, 62.0% men, 0.6% unknown) participated. This corresponds to a return rate of 45%. Median age was 64 years (17–87 years). Of all participants, 15.2% used CAM during their oncological therapy; 32.7% have used it in the past. The majority (81.9%) was not using CAM during therapy; 55.5% have not used CAM in the past respectively. The analysis revealed a significant correlation between education and CAM use during therapy (r = 0.18; p = 0.02), and CAM use in the past (r = 0.17; p = 0.04). Of all patients using CAM during therapy, favored methods were food supplements (42.3%), vitamins/minerals (42.3%), massage (34.6%). Motivations are especially the reduction of side effect and stress, the positive effect of certain CAM-treatments on the immune system and tumor therapy. Results showed no difference between women and men. Most patients not having had any experience with CAM complain about the deficiency of information by their treating oncologist (31.4%) as well as missing treatment possibilities (54.3%).

**Conclusion:**

Since many patients believe in study results demonstrating the efficacy of CAM, it stresses our task to develop innovative study protocols to investigate the outcomes of certain CAM on symptom reduction or other endpoints. Thus, prospective trials and innovative evidence-based treatment concepts to include CAM into high-end oncology is what patients demand and what a modern oncology center should offer.

## Background

When diagnosed with cancer, patients as well as their families are in immense distress; their life is filled with fear and worries. Many patients start searching for effective treatments, taking into account all possibilities offered by medicine, research, and technology. Often, the fear of treatment-related side effects drives the search for complementary and alternative medicine (CAM) options. Although data on the effect of standardized oncology is clear, patients often move to CAM and in some cases even turn away completely from Western medicine. As modern oncologists, we must keep in mind the patients' interest and expectations, and offer individualized and well-tolerated therapies in terms of standardized high-end oncology coupled with supportive care including elements of CAM as a complementary treatment.

Over the years CAM, which includes acupuncture, homeopathy, naturopathy, or special dietary concepts, gained a widespread adoption and in some areas of healthcare has reached a firm role in interdisciplinary care; for certain indications some physicians and their patients consider CAM as the preferred treatment [1,2]. In oncology, these methods are also gaining further interest and the various treatment options foster this process; however, especially when the path of Western medicine is left special care has to be taken as available data are scarce and improper handling of CAM methods is occasionally present [3–9]. For selected indications and situations, clinical data are available: Acupuncture has been shown to be associated with significant benefit compared to standard treatments in patients with lower back pain, headache or nausea [10–13]. A large German study group performing acupuncture trials (GERAC-Studies) revealed that acupuncture can reduce the frequency of a migraine similar to standard medication [12,14]. In patients with chronic back or knee pain acupuncture leveled down pain more effectively than any recommended treatment from established guidelines [15]. For many other aspects, no data or only small datasets are available, neither for acupuncture nor for other methods of CAM. In the field of oncology, smaller studies have shown beneficial effects of acupuncture to diminish fatigue, nausea, dysphagia, or other symptoms during and after oncology treatments [11–13,16].

Throughout most oncological therapies, patients suffer from side effects. Patients complain of tiredness and fatigue, loss of appetite, skin problems or headaches. The search for effective supportive care is a continuous process, also by patients themselves and their families. They choose methods of CAM to reduce their symptoms, strengthen their immune system, or follow their belief that CAM might help to cure the underlying disease.

Only few numbers are available on how many patients prefer CAM or would be open-minded to CAM treatments if offered to them when seeking medical attention. Especially in a university-based setting, where critical and skeptical voices are present against CAM, no information is available on how many patients apply CAM (in parallel) or would accept CAM when offered to them.

During setup and certification of our Oncology Center (Onkologisches Zentrum (OZ) am RHCCC am MRI TU Munich (TUM)) we have analyzed the use and acceptance of CAM scientifically. In the Munich metropolitan area, a large variety of therapists, homeopaths, physicians and others offers CAM on different levels. Numerous opportunities arise outside the university-based medicine. The aim of the present work is to understand if and which patients would be open-minded to CAM use parallel to their oncological treatment, and would favor individualized CAM within the hospital setting. Moreover, we sought to determine which methods summarized under CAM are most accepted by patients, independently of any scientific rationale, and which are the primary motivators for patients to opt for CAM.

## Materials and Methods

The National Center for Complementary and Alternative Medicine (NCCAM) has summarized different methods in complementary medicine and has sorted them into different categories [17]. Following this categorization system, we developed a patient-oriented questionnaire for oncological patients. This standardized basis generates data that can be compared with previously published data from other centers and other cultural backgrounds. All questions were tailored to fit oncology patients, and focus not only on different CAM methods but also on previous experiences with CAM, willingness to apply or use CAM when offered in a university-based setting. Development of the questionnaire included a pre-survey of 15 patients to optimize format and wording and to eliminate any difficulties in understanding the content of the single questions. The final questionnaire included 18 questions, of which some have several subitems (S1 file).

The survey was performed within the Oncology Center (Onkologisches Zentrum (OZ) am RHCCC am MRI TU Munich (TUM)) in all certified units. Since the questionnaire was handed out to all patients during a three-month time frame between May and July 2015, patients filled out the questionnaire anonymously, hence, no written consent was required by each patient. Inclusion criteria for participation were age older than 18 years, German-speaking, physical and mental ability to fill out a structured questionnaire. The Ethics Committee of the Technical University of Munich (TUM) approved the nature and content of the study with the project number 267/15.

The questionnaire mainly focuses on the following aspects: Which methods classified as CAM are most popular? Have patients been treated with CAM in the past and have had a benefit from it? Are there methods patients hesitate to accept? Which methods would patients prefer to be offered during their oncological treatment? Especially, we want to understand how the standard oncology spectrum could be expanded for oncological patients, and how many resources would they be willing to invest in such a treatment.

The survey was conducted in the Oncology Center between April and July 2015. All centers and departments involved in the certification process at that time took part in the survey. Patients were informed about the aim of the questionnaire, participation was voluntary and anonymous. Research assistants collected the anonymized data in the institutional database. The evaluation was based primarily following the criteria of the Deutsche Krebsgesellschaft (DKG) for the certification of Oncological Centers in Germany. Hence, the data was sorted according to the tumor entities, which belong to certain tumor centers, modules or focal points within the structure of the Oncology Center Munich, including the following: Nuclear medicine and radiation oncology as central units (CEN); Dermatooncology (DERMA) and Urology/Prostate (URO) representing tumor centers; Hematooncology (HEM), Endocrine malignoma (ENDO) and gastrointestinal surgery (SUR) representing focal points; as well as Neurooncology (NEURO) and head-and-neck tumors (HAN) representing a module of the Oncology Center. All patients included were treated within one of these units.

Statistical calculations were performed using SPSS Statistics v23 (IBM, USA) in a primarily descriptive way. For calculating the differences in the groups of gender, age, family status, education and monthly income nonparametric testing with the Kruskal-Wallis test was used. Pearson correlations were calculated for CAM use before and after treatment to predict the variables contributing to CAM use. A p-value ≤ 0.05 was considered as statistically significant.

## Results

A total of 171 of 376 patients (37.4% women, 62.0% men, 0.6% unknown) from seven units participated in the survey. This corresponds to a return rate of 45%. Median age was 64 years (17–87 years). For a detailed patient distribution see Table 1; for a detailed patient socio-demographic characteristics see Table 2.

**Table 1:** Patient distribution according to the participating oncological units.

Unit	Patients, n (%)	Gender		Median age (range) [years]
		Female	Male	
all	171 (100%)	37.4%	62.0%	64 (17–87)
CEN	26 (15.2%)	50.0%	46.2%	59 (29–80)
DERMA	11 (6.4%)	27.3%	72.7%	71 (33–79)
ENDO	11 (6.4%)	72.7%	27.3%	37(17–71)
HEM	24 (14.0%)	50.0%	50.0%	60 (30–80)
NEURO	37 (21.6%)	51.4%	48.6%	66 (28–87)
SUR	25 (14.6.)	36.0%	64.0%	62 (39–76)
URO	37 (21.6%)	0%	100%	68 (54–77)

**Table 2:** Patient socio-demographic characteristics of the 171 participants.

Diagnosis	Patients, n (%)
Prostate cancer	39 (22.8%)
Lung cancer	16 (9.4%)
Upper gastrointestinal cancer	13 (7.6%)
Hepato-pancreato-biliary cancer	12 (7.0%)
Lower gastrointestinal cancer	13 (7.6%)
Hematological cancer	8 (4.7%)
Brain tumors	13 (7.6%)
Skin cancer	11 (6.4%)
Thyroid cancer	10 (5.8%)
Bone / Spine cancer	11 (6.4%)
Other	17 (9.9%)
Unknown	8 (4.7%)
Received therapy ^a^	
Chemotherapy	40 (23.4%)
Radiation therapy	33 (19.3%)
Hormonal therapy	1 (0.6%)
Surgery	98 (57.3%)
Other	16 (9.4%)
Unknown	19 (11.1%)
Insurance status	
Government insurance	41 (70.8%)
Privately insured	125 (24.0%)
Unknown	5 (2.3%)
Marital status	
Single	17 (9.9%)
Married/in a relationship	125 (73.1%)
Divorced/separated Widowed	9 (5.3%)
Unknown	1 (0.6%)
Children	
Yes	124 (72.5%)
No	44 (25.7%)
Unknown	3 (1.8%)
Educational level	
Secondary (High) school 9 years	44 (25.7%)
Secondary (High) school 10 years	55 (32.2%)
Secondary (High) school 12–13 years	13 (7.6%)
College / University	52 (30.4%)
Other	2 (1.2%)
Unknown	5 (2.9%)
Monthly income (€)	
<1000	25 (14.6%)
1000–2000	49 (28.7%)
2000–3000	33 (19.3%)
3000–5000	11 (6.4%)
>5000	14 (8.2%)
Unknown	39 (22.8%)

^a^ multiple answers were possible

**Fig 1:**
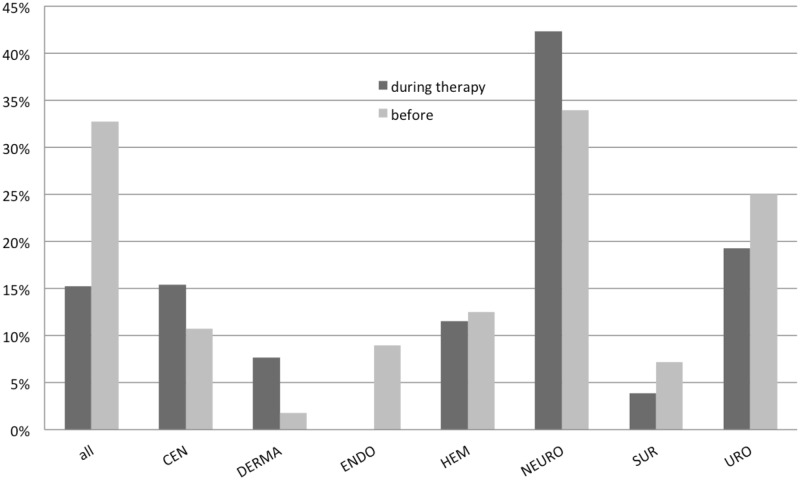
User rate of CAM. User rate of CAM during and before therapy. The percentage for all are calculated with n = 171; for the different units, the percentage of n = 26 / n = 56 are displayed for during and before therapy respectively.

Of all participants, 15.2% (26/171) used CAM during their oncological therapy (12 women/14 men); 32.7% (56/171; 26 women/30 men) have used it in the past. A difference between the participating oncological units could be demonstrated (Fig 1): patients from the units neurooncology (NEURO) and urology/prostate (URO) use CAM the most.

The majority 81.9% (140/171) of patients was not using CAM during therapy; 55.5% (95/171) have not used CAM in the past respectively. The most common reasons for rejection are the following (multiple answers were possible): for 54.3% (76/140) of patients CAM was not offered by their physician; 17.9% (25/140) had no interest in adding CAM to their therapy; 31.4% (44/140) had not enough information about CAM. This corresponds with the fact that only 26 patients (15.2%, 26/171) talked with their physician about CAM methods.

We statistically analyzed CAM use by groups of gender, age, family status, education and monthly income, and we could not prove any significant differences within the groups. However, the analysis revealed a significant correlation between education and CAM use during therapy (r = 0.18; p = 0.02), and CAM use in the past (r = 0.17; p = 0.04).

**Fig 2:**
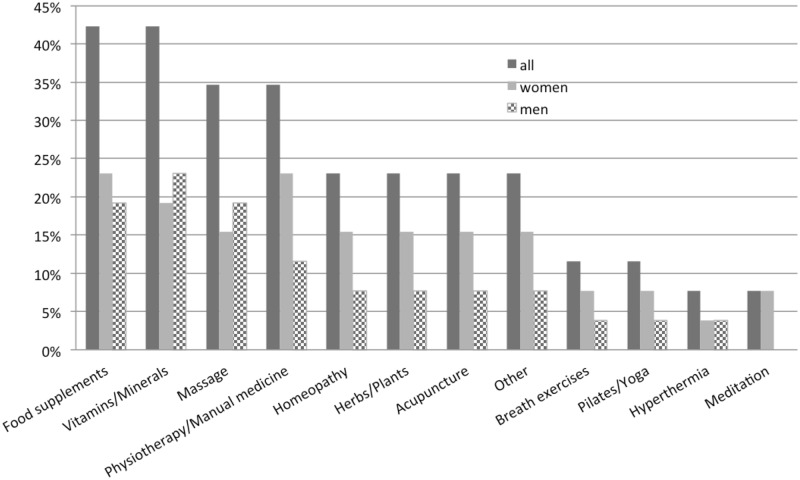
User rate of CAM. User rates of CAM methods during oncological therapy (n = 26).

**Fig 3:**
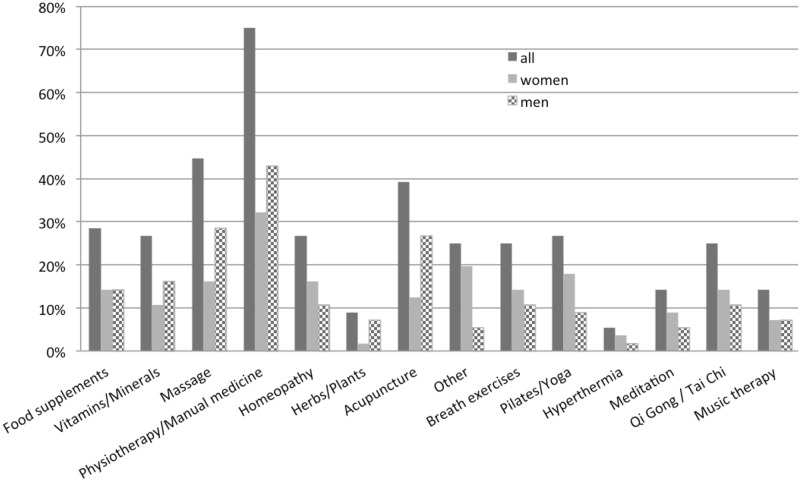
User rate of CAM. User rates of CAM methods before oncological therapy (n = 56).

Of all patients using CAM during therapy, the most applied methods were food supplements (42.3%) and vitamins/minerals (42.3%) as well as massage (34.6%) and physiotherapy/manual medicine (26.9%) followed by homeopathy (23.1%) and herbs/plants (23.1%). Fig 2 summarizes the user rates of all CAM methods, also divided by gender. Apart from vitamins, massage, and hyperthermia, women represent the majority using CAM. Moreover, the effect of the CAM treatment was rated as good (46.2%, 12/26), moderate (19.2%, 5/26) and uncertain (34.6%, 9/26). Only one patient eating food supplements reported side effects by using CAM caused by diabetes. In comparison, Fig 3 shows the user rates of CAM methods used in the past.

Patients found out about CAM (multiple answers possible) by their treating physician/therapist (50.0%; 48.2% during and before therapy, respectively) or oncologist (23.1%; 3.5%), by self-research (23.1%; 30.3%) and through recommendations of family/friends (34.6%; 50.0%).

**Fig 4:**
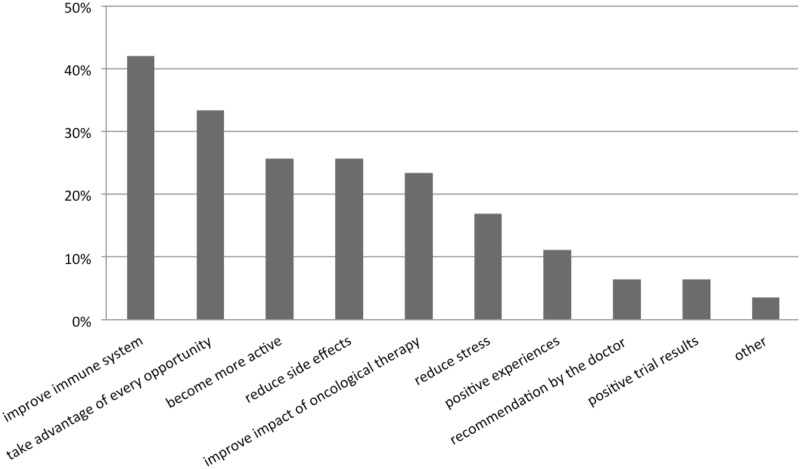
User motives. Motives of oncological patients to use CAM (n = 171).

All patients (n = 171) stated their motives for using CAM during therapy or for possibly using it in the future: Primarily to improve the immune system (42.1%, 72/171) and to take advantage of every opportunity (33.3%, 57/171) as well as to reduce therapy side effects (25.7%, 44/171), become more active (25.7%, 44/171) and the assumption of improving the impact of the oncological therapy (23.4% 40/171) by using CAM (Fig 4). Results showed no difference between women and men.

Patients suggested improving the information about CAM for supporting oncological therapies by including personal consultations by specialists during their treatment period (49.1%, 84/171), offering flyer/brochures (29.2%, 50/171) and providing information on the department/clinic homepage (16.4%, 28/171). Among all respondents, 40.9% (70/171) would bear the costs for a concomitant CAM treatment integrated into their oncological therapy if the health insurance would not pay. Consequently, if a CAM method would be offered as concomitant therapy, 54.4% (93/171) patients would be willing to add it to their treatment.

## Discussion

The present study evaluates the attitude towards CAM in a university-based Oncology Center. We analyzed answers from 171 patients and the results show that about 15–33% of all patients in oncology have used CAM in the past, and used CAM in parallel to their standard treatment in the current situation. Differences between tumor entities can be observed. Especially patients with neuro-oncological diagnoses have a strong affinity to CAM. Moreover, independently of the underlying cancer, about 41% of patients in favor of CAM are willing to invest a certain amount of money should the treatment not be covered by their health insurance.

The use of complementary and alternative medicine is rising worldwide, not only in Western countries, but also in the Far East or in third-world countries, and it is becoming increasingly popular in cancer patients worldwide [18–33]. Previous reports have shown that in certain countries, such as the United States of America, up to 91% of cancer patients are applying or are being treated with at least one form of CAM [34]. In Asia, where many techniques such as herbal therapies, acupuncture, moxibustion, or Qi Gong have been popular for centuries, about 45% of all cancer patients are treated with some kind of CAM [35]. In Europe, a multi-institutional study within 14 countries revealed that approximately 36% of all cancer patients apply CAM, depending on the country between 15% to 73% [6]. The reasons for the increasing interest in CAM are diverse. Compared to non-CAM-users the typical CAM-using patient is thought to be exposed to high psychological distress and perhaps reduced quality of life; CAM use has been reported to be associated with depression, anxiety, fear of tumor recurrence but also weak mental health [36–38]. Furthermore, studies have shown that the use of CAM is associated with higher education or high socioeconomic status [25,39–42], which we could also show in our results. Women are significantly more often open to CAM than men, independently of the racial background [25,39]. Most studies have shown that either younger patients <40 years of age or retirees significantly more often use CAM [25]. However, some studies have not confirmed this higher prevalence in women or in patients with higher education [43].

In our study, the most preferred methods of CAM were food supplements (42.3%) and vitamins/minerals (42.3%) as well as massage (34.6%) and physiotherapy/manual medicine (26.9%) followed by homeopathy (23.1%) and herbs/plants (23.1%). This is in line with the literature: Abdallah and colleagues described a frequent utilization of vitamins/minerals as well as herbs in a group of women with gynecological malignancies [25]; Nazik et al. reported 90.2% of all patients in their study favoring herbal therapy [44]. For the subgroup of head-and-neck cancer patients, Molassiotis et al. showed that 47.1% of all patients chose herbal medicine, followed by medicinal teas (23.5%) or vitamins/minerals (11.8%) [5]. Within the large European study on 956 patients with various diagnoses also homeopathy, medicinal teas, and vitamins/minerals were the most frequently used CAM methods [6].

Eventually, there are several patient-specific reasons why patients choose CAM. One aspect is the common belief that different methods of CAM have the potential to “boost”the immune system and to strengthen the body to fight cancer. This was revealed in different studies and consistent with our results (Fig 4): Yildirim et al. observed that modulation of the immune system was the main argument for the use of CAM in a Turkish group of patients with gynecological cancer [45]. A European survey on the use of CAM reported that over 50% of all patients were using CAM to increase their body’s ability to fight the disease [6].

Importantly, there is some evidence that the interaction between certain methods of CAM and chemotherapy or radiation might counteract their efficacy: The production of free radicals by cancer treatment, which is thought to be an essential part of the treatment efficacy, could potentially antagonize by the antioxidant effect of some supplements. However, preclinical and clinical evaluations have led to inconsistent data [46–52].

## Conclusion

About one-third of all patients have had experience with CAM in the past and are open to the possibility to include CAM into their standard oncological treatment. Motivations are especially the reduction of side effect and stress, the positive effect of certain CAM-treatments on the immune system and tumor therapy. Most patients not having had any experience with CAM complain about the deficiency of information by their treating oncologist as well as missing treatment possibilities. This underlines the necessity to evaluate CAM in a university-based setting to determine which options have efficacy and which do not; moreover, this might have the potential to offer evidence-based CAM as a complementary treatment to high-end oncology care. Since many patients believe in study results demonstrating the efficacy of CAM, which is somewhat controversial in the existing literature, it stresses our task to develop innovative study protocols to demonstrate the positive effect of certain CAM on symptom reduction or other endpoints. Currently, in our Department of Radiation Oncology, we are conducting a prospective trial to evaluate the effect of acupuncture to reduce radiotherapy-related side effects (ROSETTA-Trial). Thus, prospective trials and innovative evidence-based treatment concepts to include CAM into high-end oncology is what patients demand and what a modern oncology center should consider offering.

## Supporting Information

S1 File CAM questionnaire. Questionnaire about complementary and alternative medicine handed out to all patients of the Munich Oncology Center.
(PDF)

## References

[1] Horneber M, Bueschel G, Dennert G, Less D, Ritter E, Zwahlen M. How Many Cancer Patients Use Complementary and Alternative Medicine: A Systematic Review and Metaanalysis. Integr Cancer Ther. 2012;11: 187–203. 10.1177/1534735411423920 22019489

[2] Nissen N, Schunder-Tatzber S, Weidenhammer W, Johannessen H. What Attitudes and Needs Do Citizens in Europe Have in Relation to Complementary and Alternative Medicine? Forsch Komplementmed. 2012;19: 9–17. 10.1159/000342710 23883940

[3] Huebner J, Münstedt K. Alternative therapies in oncology. Onkologe. 2009 10.1007/s00761-009-1764-3

[4] Huebner J, Prott FJ, Micke O, Muecke R, Senf B, Dennert G, et al Online survey of cancer patients on complementary and alternative medicine. Oncol Res Treat. Karger Publishers; 2014;37: 304–308. 10.1159/000362616 24903760

[5] Molassiotis A, Ozden G, Platin N. Complementary and alternative medicine use in patients with head and neck cancers in Europe. European Journal of …. 2006 10.1111/j.1365-2354.2005.00615.x/pdf 16441673

[6] Molassiotis A, Fernadez-Ortega P, Pud D, Ozden G, Scott JA, Panteli V, et al Use of complementary and alternative medicine in cancer patients: a European survey. 2005;16: 655–663. 10.1093/annonc/mdi110 15699021

[7] Rausch SM, Winegardner F, Kruk KM, Phatak V, Wahner-Roedler DL, Bauer B, et al Complementary and alternative medicine: use and disclosure in radiation oncology community practice. Supportive Care in Cancer. 2010;19: 521–529. 10.1007/s00520-010-0846-5 20336329

[8] Robotin M, Holliday C, Bensoussan A. Defining research priorities in complementary medicine in oncology. 2012;20: 345–352. 10.1016/j.ctim.2012.04.001 22863650

[9] Micke O, Bruns F, Glatzel M, Schönekaes K, Micke P, Mücke R, et al Predictive factors for the use of complementary and alternative medicine (CAM) in radiation oncology. European Journal of Integrative Medicine. 2009;1: 19–25.

[10] Vickers A, Rees R, Zollman C, McCarney R, Smith C, Ellis N, et al Acupuncture of chronic headache disorders in primary care: randomised controlled trial and economic analysis. Health Technol Assess. 2004;8 10.3310/hta8480 15527670

[11] Simcock R, Fallowfield L, Monson K, Solis-Trapala I, Parlour L, Langridge C, et al ARIX: A randomised trial of acupuncture v oral care sessions in patients with chronic xerostomia following treatment of head and neck cancer. 2013;24: 776–783. 10.1093/annonc/mds515 23104718

[12] Haake M. German Acupuncture Trials (Gerac) For Chronic Low Back Pain<subtitle>Randomized, Multicenter, Blinded, Parallel-Group Trial With 3 Groups</subtitle>. Arch Intern Med. 2007;167: 1892 10.1001/Archinte.167.17.1892 17893311

[13] Enblom A, Johnsson A, Hammar M, Onelöv E, Steineck G, Börjeson S. Acupuncture compared with placebo acupuncture in radiotherapy-induced nausea—a randomized controlled study. Ann Oncol. 2012;23: 1353–1361. 10.1093/annonc/mdr402 21948812

[14] Endres HG, Zenz M, Schaub C, Molsberger A, Haake M, Streitberger K, et al German Acupuncture Trials (gerac) address problems of methodology associated with acupuncture studies. Schmerz. 2004;19: 201–213. 10.1007/s00482-004-0345-z 15959826

[15] Endres HG, Diener HC, Maier C, Böwing G. Akupunktur bei chronischen Kopfschmerzen. Dtsch …. 2007.

[16] Vickers AJ, Straus DJ, Fearon B, Cassileth BR. Acupuncture for Postchemotherapy Fatigue: A Phase II Study. J Clin Oncol. 2004;22: 1731–1735. 10.1200/JCO.2004.04.102 15117996

[17] National Institutes of Health, Bethesda, MD (US). Activities of Human Gene Nomenclature Committee. 2002 Jul. 10.2172/804185

[18] Gunawan S, Arnoldussen M, Gordijn MS, Sitaresmi MN, van de Ven PM, Broeke ten CAM, et al Comparing Health-Care Providers' Perspectives on Complementary and Alternative Medicine in Childhood Cancer Between Netherlands and Indonesia. Pediatr Blood Cancer. 2015;63: 118–123. 10.1002/pbc.25689 26274831

[19] Magi T, Kuehni CE, Torchetti L, Wengenroth L, Lüer S, Frei-Erb M. Use of Complementary and Alternative Medicine in Children with Cancer: A Study at a Swiss University Hospital. Sethi G, editor. 2015;10: e0145787 10.1371/journal.pone.0145787 26694320 PMC4687920

[20] Naja F, Fadel RA, Alameddine M, Aridi Y, Zarif A, Hariri D, et al Complementary and alternative medicine use and its association with quality of life among Lebanese breast cancer patients: a cross-sectional study. BMC Complement Altern Med. 2015;15: 102 10.1186/s12906-015-0969-9 26692096 PMC4687122

[21] Akpunar D, Bebis H, Yavan T. Use of Complementary and Alternative Medicine in Patients with Gynecologic Cancer: a Systematic Review. Asian Pac J Cancer Prev. 2015;16: 7847–7852. 10.7314/APJCP.2015.16.17.7847 26625809

[22] Mani J, Jüngel E, Bartsch G, Filmann N, Nelson K, Ackermann H, et al Use of complementary and alternative medicine before and after organ removal due to urologic cancer. PPA. 2015;: 1407 10.2147/PPA.S90061 26491269 PMC4599187

[23] Üstündağ S, Demir Zencirci A. Complementary and Alternative Medicine Use Among Cancer Patients and Determination of Affecting Factors. Holistic Nursing Practice. 2015;29: 357–369. 10.1097/HNP.0000000000000113 26465625

[24] AlBedah AM, Khalil MK. Cancer Patients, Complementary Medicine and Unmet Needs in Saudi Arabia. Asian Pac J Cancer Prev. 2015;16: 6799–6799. 26434915 10.7314/apjcp.2015.16.15.6799

[25] Abdallah R, Xiong Y, Lancaster JM, Judson PL. Complementary and Alternative Medicine Use in Women With Gynecologic Malignancy Presenting for Care at a Comprehensive Cancer Center. 2015;25: 1724–1730. 10.1097/IGC.0000000000000549 26397156

[26] Ojukwu M, Mbizo J, Leyva B, Olaku O, Zia F. Complementary and Alternative Medicine Use Among Overweight and Obese Cancer Survivors in the United States. Integr Cancer Ther. 2015;14: 503–514. 10.1177/1534735415589347 26044767

[27] Adams J, Valery P. C., Sibbritt D, Bernardes CM, Broom A, Garvey G. Use of Traditional Indigenous Medicine and Complementary Medicine Among Indigenous Cancer Patients in Queensland, Australia. Integr Cancer Ther. 2015;14: 359–365. 10.1177/1534735415583555 25953415

[28] Knight A, Hwa YS, Hashim H. Complementary Alternative Medicine Use Amongst Breast Cancer Patients in the Northern Region of Peninsular Malaysia. Asian Pac J Cancer Prev. 2015;16: 3125–3130. 10.7314/APJCP.2015.16.8.3125 25921108

[29] Sullivan A, Gilbar P, Curtain C. Complementary and Alternative Medicine Use in Cancer Patients in Rural Australia. Integr Cancer Ther. 2015;14: 350–358. 10.1177/1534735415580679 25873293

[30] Montazeri A, Sajadian A, Ebrahimi M, Akbari ME. Depression and the use of complementary medicine among breast cancer patients. Supportive Care in Cancer. 2004;13: 339–342. 10.1007/s00520-004-0709-z 15549425

[31] Montazeri A, Sajadian A, Ebrahimi M, Haghighat S, Harirchi I. Factors predicting the use of complementary and alternative therapies among cancer patients in Iran. Eur J Cancer Care. 2007;16: 144–149. 10.1111/j.1365-2354.2006.00722.x 17371423

[32] Cassileth BR, Schraub S, Robinson E, Vickers A. Alternative medicine use worldwide: the International Union Against Cancer survey. 2001;91: 1390–1393. 10.1002/1097-0142(20010401)91:7<1390::aid-cncr1143>3.0.co;2-c 11283941

[33] Asadpour R, Meng Z, Kessel KA, Combs SE. Use of acupuncture to alleviate side effects in radiation oncology—Current evidence and future directions. Advances in Radiation Oncology VL—IS—SP—EP—PY—T2. 2016 10.1016/j.adro.2016.08.002 PMC5514158 28740905

[34] Yates JS, Mustian KM, Morrow GR, Gillies LJ, Padmanaban D, Atkins JN, et al Prevalence of complementary and alternative medicine use in cancer patients during treatment. Supportive Care in Cancer. 2005;13: 806–811. 10.1007/s00520-004-0770-7 15711946

[35] Hyodo I. Nationwide Survey on Complementary and Alternative Medicine in Cancer Patients in Japan. J Clin Oncol. 2004;23: 2645–2654. 15728227 10.1200/JCO.2005.04.126

[36] Burstein HJ, Gelber S, Guadagnoli E, Weeks JC. Use of Alternative Medicine by Women with Early-Stage Breast Cancer. 1999;340: 1733–1739. 10.1056/NEJM199906033402206 10352166

[37] Risberg T, Jacobsen BK. The association between mental distress and the use of alternative medicine among cancer patients in North Norway. 2003;12: 539–544. 10.1023/a:1025063705413 13677498

[38] Correa-Velez I, Clavarino A, Barnett AG, Eastwood H. Use of complementary and alternative medicine and quality of life: changes at the end of life. Palliat Med. 2003;17: 695–703. 14694921 10.1191/0269216303pm834oa

[39] Shumer G, Warber S, Motohara S, Yajima A, Plegue M, Bialko M, et al Complementary and alternative medicine use by visitors to rural Japanese family medicine clinics: results from the international complementary and alternative medicine survey. BMC Complement Altern Med. 2014;14: 360 10.1186/1472-6882-14-360 25256591 PMC4192731

[40] Begbie SD, Kerestes ZL, Bell DR. Patterns of alternative medicine use by cancer patients. Med J Aust. 1996;165: 545–548. 8941239 10.5694/j.1326-5377.1996.tb138639.x

[41] Barnes PM, Bloom B, Nahin RL. Complementary and Alternative Medicine Use Among Adults and Children: United States, 2007. 19361005

[42] Richardson MA, Sanders T, Palmer JL, Greisinger A, Singletary SE. Complementary/alternative medicine use in a comprehensive cancer center and the implications for oncology. J Clin Oncol. 2000;18: 2505–2514. 10893280 10.1200/JCO.2000.18.13.2505

[43] Paul M, Davey B, Senf B, Stoll C, Münstedt K, Mucke R, et al Patients with advanced cancer and their usage of complementary and alternative medicine. J Cancer Res Clin Oncol. 2013;139: 1515–1522. 10.1007/s00432-013-1460-y 23832609 PMC11824496

[44] Nazik E, Nazik H, Api M, Kale A, Aksu M. Complementary and Alternative Medicine Use by Gynecologic Oncology Patients in Turkey. Asian Pac J Cancer Prev. 2012;13: 21–25. 10.7314/APJCP.2012.13.1.021 22502670

[45] Yildirim Y, Tinar S, Yorgun S, Toz E, Kaya B, Sonmez S, et al The use of complementary and alternative medicine (CAM) therapies by Turkish women with gynecological cancer. Eur J Gynaecol Oncol. 2006;27: 81–85. 16550977

[46] Nakayama A, Alladin KP, Igbokwe O, White JD. Systematic Review: Generating Evidence-Based Guidelines on the Concurrent Use of Dietary Antioxidants and Chemotherapy or Radiotherapy. Cancer Investigation. 2011;29: 655–667. 10.3109/07357907.2011.626479 22085269 PMC3666569

[47] Lawenda BD, Kelly KM, Ladas EJ, Sagar SM, Vickers A, Blumberg JB. Should Supplemental Antioxidant Administration Be Avoided During Chemotherapy and Radiation Therapy? JNCI Journal of the National Cancer Institute. 2008;100: 773–783. 10.1093/jnci/djn148 18505970

[48] Simone CB, Simone NL, Simone V. Antioxidants and other nutrients do not interfere with chemotherapy or radiation therapy and can increase kill and increase survival, part 1. Altern Ther Health Med. 2007;13: 22–28. 17283738

[49] Simone CB, Simone NL, Simone V, Simone CB. Antioxidants and other nutrients do not interfere with chemotherapy or radiation therapy and can increase kill and increase survival, Part 2. Altern Ther Health Med. 2007;13: 40–47. 17405678

[50] D'Andrea GM. Use of Antioxidants During Chemotherapy and Radiotherapy Should Be Avoided. CA Cancer J Clin. 2005;55: 319–321. 10.3322/canjclin.55.5.319 16166076

[51] Labriola D, Livingston R. Possible interactions between dietary antioxidants and chemotherapy. 1999;13: 1003–8– discussion 1008–1011–2. 10442346

[52] Yeung KS, Gubili J. Clinical Guide to Herb-Drug Interactions in Oncology. J Soc Integr Onco. 2007;05: 113. 10.2310/7200.2007.008 17761130

