# Supplementary material for: Use of Complementary and Alternative Medicine (CAM) as Part of the Oncological Treatment: Survey about Patients’ Attitude towards CAM in a University-Based Oncology Center in Germany
Source: PLoS One. 2016 Nov 3;11(11):e0165801. doi: 10.1371/journal.pone.0165801 (PMC5094772; doi:10.1371/journal.pone.0165801)
Supplement: S1 File — Questionnaire about complementary and alternative medicine handed out to all patients of the Munich Oncology Center. (PDF) [file pone.0165801.s001.pdf]

Klinikum rechts der Isar
Technische Universität München

Klinikum rechts der Isar
Anstalt des öffentlichen Rechts

Onkologisches Zentrum
Am RHCCC im MRI TU München
Ismaninger Straße 22
81675 München

Univ.-Prof. Dr. med. S. E. Combs
Leiterin des Onkologischen Zentrums

Fragebogen zu Komplementärmedizinischen Therapien

Sehr geehrte Patientin, sehr geehrter Patient,

komplementärmedizinische Therapien, beispielweise Naturheilverfahren, spezielle Ernährungs-methoden, Akupunktur, Homöopathie oder Hydrotherapie wie Kneippsche Anwendungen, sind weit verbreitet. Für einige dieser Behandlungen gibt es klinische Studien, die einen Vorteil belegen. Bei vielen Methoden ist jedoch die Datenlage sehr dünn oder nicht vorhanden. Sie werden aber dennoch häufig verschrieben und durchgeführt.

In den letzten Jahren ist die Nachfrage nach komplementärmedizinischen Therapien deutlich gestiegen. Viele Ärzte bieten neben der traditionellen Schulmedizin komplementärmedizinische Therapien an.

Um besser zu verstehen, welche dieser Methoden bei unseren Patienten am beliebtesten sind, welche vielleicht in der Vergangenheit schon eine Symptomlinderung oder einen anderen Effekt gezeigt haben, oder welche der Therapien auf Zurückhaltung bei unseren Patienten stoßen, haben wir einen Fragebogen zusammengestellt.

Wir würden Sie bitten, diesen Fragebogen so genau wie möglich zu beantworten. Ihre Antworten werden anonymisiert behandelt. Die gemachten Angaben haben keinen Einfluss auf Ihre weitere onkologische Behandlung. Sollten Sie diesen Fragebogen nicht beantworten wollen, entsteht Ihnen kein therapeutischer Nachteil.

Für Rückfragen stehen mein Team und ich Ihnen immer gerne zur Verfügung.

Mit den besten Wünschen für Ihre Genesung,

Ihre

Stephanie E. Combs

Vorstand:
Univ.-Prof. Dr. Reiner Gradinger
(Ärztlicher Direktor, Vorsitzender)
Markus Zendler
(Kaufmännischer Direktor)
Anette Thoke-Colberg
(Pflegedirektorin)
Univ.-Prof. Dr. Peter Henningsen
(Dekan)

Bankverbindung:
Bayer. Landesbank Girozentrale
Kto-Nr. 20 272
BLZ 700 500 00

BIC: BYLADEMM
IBAN: DE82 7005 0000 0000 0202 72
USt-IdNr. DE 129 52 3996

Nr. _____

1. Ihr Geschlecht **Ihr Alter**

☐ weiblich ☐ männlich ☐ _______Jahre

2. Ihr Familienstatus:

☐ Single ☐ in einer Partnerschaft ☐ verheiratet

☐ verwitwet ☐ geschieden/getrennt

3. Haben Sie Kinder?

☐ ja ☐ nein

Wenn ja, wie alt? ____

4. Ihre höchste schulische Ausbildung:

☐ Hauptschule ☐ Realschule ☐ Gymnasium

☐ Universität/Fachhochschule ☐ andere: ______________________________

5. Ihr monatliches Einkommen (Netto) beträgt

☐ <1000 Euro ☐ 1000 – 2000 Euro ☐ 2000 – 3000 Euro

☐ 3000 – 5000 Euro ☐ >5000 Euro

6. Ihre Krankenversicherung

☐ gesetzlich ☐ privat

7. Für welche Krebserkrankung werden Sie derzeit bei uns behandelt?
(Mehrfachantworten möglich)

☐ Brust ☐ Prostata ☐ Hodentumor ☐ Harnblase ☐ Lunge

☐ Niere ☐ Leber ☐ Lymphom ☐ Speiseröhre ☐ Bauchspeicheldrüse

☐ Gehirn ☐ Knochen ☐ Haut ☐ Darm ☐ Hals/Nase/Ohren

☐ Gynäkologischer Tumor ☐ andere: ____________________________

8. Sie erhalten bei uns eine

☐ Chemotherapie ☐ Strahlentherapie ☐ OP ☐ Hormontherapie

☐ Strahlentherapie + Chemotherapie

☐ andere: ______________________________

9. Nehmen Sie derzeit Medikamente ein?

☐ ja ☐ nein

Wenn ja, welche? ____

____

10. Werden Sie derzeit, parallel zu Ihrer onkologischen Therapie mit einer komplementärmedizinischen Therapie behandelt?

☐ ja ☐ nein

a) Wenn nein, warum können bzw. wollen Sie keine komplementärmedizinische Therapie in Anspruch nehmen? (Mehrfachantworten möglich)

☐ wurde mir nicht angeboten ☐ kein Interesse ☐ zu wenig Information
☐ schlechte Erfahrung ☐ andere: ______________________________

b) Wenn ja, welche? (Mehrfachantworten möglich)

☐ Vitamine/Mineralstoffe ☐ Kräuter ☐ Nahrungsergänzungsmittel
☐ Massage ☐ Chiropraktik ☐ Pilates
☐ Osteopathie ☐ Physiotherapie ☐ Meditation
☐ Tai Chi ☐ Akupunktur ☐ Qi Gong
☐ Yoga ☐ Musik-/Kunsttherapie ☐ Atmungsübungen
☐ Ozontherapie ☐ Lichttherapie ☐ Homöopathie
☐ Hydrotherapie ☐ Hypnotherapie ☐ Ayurveda
☐ Hyperthermie ☐ Traditionelle Chinesische Medizin (TCM)
☐ andere: ______________________________

c) Wenn ja, durch wen wird diese Behandlung durchgeführt?

☐ Hausarzt/-ärztin ☐ Onkologe/-in ☐ Heilpraktiker/-in
☐ andere: ______________________________

d) Wenn ja, wie sind Sie auf diese Therapie aufmerksam geworden?
(Mehrfachantworten möglich)

☐ selbst recherchiert (Internet, Zeitung...) ☐ Empfehlung durch den/die Hausarzt/-ärztin
☐ Empfehlung anderer Krebspatienten ☐ Empfehlung durch den/die Onkologen/-in

☐ Empfehlung durch Familie, Freunde, Bekannte

☐ andere: ____

e) Wenn ja, wie wirken die von Ihnen verwendeten komplementärmedizinischen Therapien?

☐ guter Effekt ☐ mittelmäßiger Effekt ☐ kein Effekt ☐ ungewiss

f) Wenn ja, haben Sie aufgrund der von Ihnen verwendeten komplementärmedizinischen Therapien Nebenwirkungen?

☐ ja ☐ nein ☐ ungewiss

Wenn ja, welche? ____

____

11. Warum nehmen bzw. würden Sie gerne komplementärmedizinische Therapien in Anspruch nehmen? (Mehrfachantworten möglich)

☐ um das Immunsystem zu verbessern

☐ um keine Chance/Möglichkeit auszulassen

☐ um die Nebenwirkungen der Therapie zu verringern

☐ um aktiver zu werden

☐ wegen positiver Erfahrungen

☐ um Stress zu reduzieren

☐ um eine bessere Wirkung der onkologischen Therapie zu erlangen

☐ wegen persönlicher Empfehlung durch einen Arzt

☐ wegen Studienergebnissen, die besagen, dass Komplementärmedizin einen Behandlungsvorteil hat

☐ andere: ____

12. Haben Sie mit Ihrer Ärztin/Ihrem Arzt über Ihre aktuellen komplementärmedizinischen Therapien oder allgemein über diese Methoden gesprochen? (Mehrfachantworten möglich)

ja, weil

☐ der Arzt danach gefragt hat

☐ der Arzt darüber informiert sein sollte

☐ ich die Meinung des Arztes wissen wollte

☐ andere: __________________________

nein, weil

☐ der Arzt nicht danach gefragt hat

☐ es nicht wichtig ist für den Arzt

☐ der Arzt nicht einverstanden sein könnte

☐ andere: __________________________

13. Falls Sie mit Ihrer Ärztin/Ihrem Arzt darüber gesprochen haben, wie war ihre/seine Reaktion?

____

____

14. Haben Sie in der Vergangenheit schon einmal komplementärmedizinische Therapien in Anspruch genommen?

☐ ja ☐ nein

a) Wenn ja, welche? (Mehrfachantworten möglich)

☐ Vitamine/Mineralstoffe ☐ Kräuter ☐ Nahrungsergänzungsmittel
☐ Massage ☐ Chiropraktik ☐ Pilates
☐ Osteopathie ☐ Physiotherapie ☐ Meditation
☐ Tai Chi ☐ Akupunktur ☐ Qi Gong
☐ Yoga ☐ Musik-/Kunsttherapie ☐ Atmungsübungen
☐ Ozontherapie ☐ Lichttherapie ☐ Homöopathie
☐ Hydrotherapie ☐ Hypnotherapie ☐ Ayurveda
☐ Hyperthermie ☐ Traditionelle Chinesische Medizin (TCM)
☐ andere: ______________________________

b) Wenn ja, aus welchem Grund haben Sie diese Behandlung durchgeführt?

____

c) Durch wen wurde diese Behandlung durchgeführt?

☐ Hausarzt/-ärztin ☐ Onkologe/-in ☐ Heilpraktiker/-in
☐ andere: ______________________________

d) Wie sind Sie auf diese Behandlung aufmerksam geworden?

☐ selbst recherchiert (Internet, Zeitung...) ☐ Empfehlung durch den/die Hausarzt/-ärztin
☐ Empfehlung anderer Krebspatienten ☐ Empfehlung durch den/die Onkologen/-in
☐ Empfehlung durch Familie, Freunde, Bekannte
☐ andere: ______________________________

15. Welches Informationsmaterial würden Sie sich über komplementärmedizinische Therapien wünschen? (Mehrfachantworten möglich)

☐ persönliches Beratungsgespräch ☐ Flyer/Broschüren

☐ Informationen auf der Homepage der Abteilung

☐ andere: ____

16. Wenn Ihre Krankenkasse die Behandlung mit komplementärmedizinischen Methoden nicht bezahlen würde, wären Sie bereit die Kosten hierfür selbst zu übernehmen?

☐ ja ☐ nein

a) Wenn ja, was würden Sie monatlich dafür ausgeben? ________________

b) Was haben Sie in der Vergangenheit monatlich dafür ausgegeben? ______________

17. Wenn komplementärmedizinische Therapien in der onkologischen Therapie angeboten werden würden, wie viele zusätzliche Termine pro Woche bzw. wie viel Zeit hätten Sie dafür zur Verfügung?

____

18. Würden Sie sich durch uns parallel zur onkologischen Therapie mit komplementärmedizinischen Methoden (z.B. Akupunktur) behandeln lassen?

☐ ja ☐ nein

Kommentare

*** Wir bedanken uns bei Ihnen für das Ausfüllen des Fragebogens! ***
